# Dorsolateral prefrontal lesions do not impair tests of scene learning and decision-making that require frontal–temporal interaction

**DOI:** 10.1111/j.1460-9568.2008.06353.x

**Published:** 2008-08

**Authors:** Mark G Baxter, David Gaffan, Diana A Kyriazis, Anna S Mitchell

**Affiliations:** Department of Experimental Psychology, Oxford UniversitySouth Parks Road, Oxford OX1 3UD, UK

**Keywords:** episodic, frontal cortex, macaque, memory, strategy

## Abstract

Theories of dorsolateral prefrontal cortex (DLPFC) involvement in cognitive function variously emphasize its involvement in rule implementation, cognitive control, or working and/or spatial memory. These theories predict broad effects of DLPFC lesions on tests of visual learning and memory. We evaluated the effects of DLPFC lesions (including both banks of the principal sulcus) in rhesus monkeys on tests of scene learning and strategy implementation that are severely impaired following crossed unilateral lesions of frontal cortex and inferotemporal cortex. Dorsolateral lesions had no effect on learning of new scene problems postoperatively, or on the implementation of preoperatively acquired strategies. They were also without effect on the ability to adjust choice behaviour in response to a change in reinforcer value, a capacity that requires interaction between the amygdala and frontal lobe. These intact abilities following DLPFC damage support specialization of function within the prefrontal cortex, and suggest that many aspects of memory and strategic and goal-directed behaviour can survive ablation of this structure.

## Introduction

The dorsolateral prefrontal cortex (DLPFC), including area 46 (Petrides & Pandya, 1999), is thought to be critical for many higher-order and supervisory aspects of cognitive function. These have variously been described in terms of working memory (Goldman-Rakic, 1996), monitoring of stimuli in memory (Petrides, 1995; Petrides *et al.*, 2002), attentional selection (Passingham, 1993; Rowe & Passingham, 2001), making flexible decisions (Heekeren *et al.*, 2006; Seo *et al.*, 2007) and in temporal organization of behaviour (Fuster, 1990, 1991).

DLPFC itself receives only weak direct input from inferotemporal cortex and amygdala (Kawamura & Naito, 1984; Barbas & De Olmos, 1990; Petrides & Pandya, 1999, 2002). However, it is substantially interconnected with other subregions of prefrontal cortex (Kawamura & Naito, 1984; Petrides & Pandya, 1999, 2002), and so is placed to modulate and interact with other areas of prefrontal cortex that do receive substantial direct inputs from the temporal lobes (Webster *et al.*, 1994; Carmichael & Price, 1995; Petrides & Pandya, 2002). The motivation for the current study was to examine the effects of damage limited to DLPFC on the performance of a number of tasks that require interaction between the frontal cortex and the temporal lobes. Based on the presence of neurophysiological signatures within DLPFC for cognitive processes that are engaged by these tasks, we considered that DLPFC may be involved in performance of three tests of visual memory and decision-making that require frontal–temporal interaction. Although these tasks are vulnerable to damage to other subregions of the prefrontal cortex (Izquierdo *et al.*, 2004; Baxter *et al*., 2007, 2008; Wilson *et al.*, 2007; M.G. Baxter, D. Gaffan, D.A. Kyriazis and A.S. Mitchell, unpublished observations), a number of observations support a potential role for the DLPFC in performance of each task as well.

The first task is object-in-place scene learning, which has been described as a monkey model of episodic memory (Gaffan, 1994) because monkeys learn new object-in-place problems extremely rapidly as a consequence of their being embedded in unique background scenes that provide a context for learning. Scene learning is dramatically impaired by unilateral lesions of frontal cortex in one hemisphere (including DLPFC as well as medial, ventrolateral and orbital prefrontal cortex) and the inferotemporal cortex in the other, preventing intrahemispheric communication between these structures (Browning *et al.*, 2005). DLPFC activation, observed as increased blood-oxygen-level-dependent (BOLD) signal in functional magnetic resonance imaging experiments, associated with memory encoding strategies (Bor *et al.*, 2003) and episodic encoding and recognition (Ranganath *et al.*, 2003; Murray & Ranganath, 2007) suggests that DLPFC damage may impair scene learning. The second task is a test of strategy implementation that requires monkeys to alternate choices between two categories of visual objects, each associated with a different reward schedule that results in delivery of food reward. This requires active monitoring of choices, appropriate temporal sequencing of behaviour and the implementation of well-learned behavioural rules in order to optimize the rate of reward delivery, capacities that are associated with activity of neurons recorded in the DLPFC (Robbins, 1996; White & Wise, 1999; Hoshi & Tanji, 2004; Lee & Seo, 2007; Seo *et al.*, 2007). Performance in this strategy implementation task is also disrupted by frontal-inferotemporal disconnection (Gaffan *et al.*, 2002). The third task is a test of value-based decision-making in a reinforcer devaluation procedure. The monkey is allowed to choose between objects that lead to different reward outcomes (different foods). The value of the food rewards is manipulated by a reversible satiation manipulation, which results in changes in choice behaviour in normal monkeys but not in monkeys with disruption of amygdala, orbital prefrontal cortex or communication between them (Málková*et al.*, 1997; Baxter *et al.*, 2000; Izquierdo *et al.*, 2004). Conjoint representation of reward and response information by single neurons in DLPFC (Leon & Shadlen, 1999; Wallis & Miller, 2003) as well as DLPFC involvement in decision-making (Lee & Seo, 2007) would suggest that DLPFC damage could impair choice behaviour in this task as well.

## Materials and methods

### Subjects

Seven rhesus monkeys (*Macaca mulatta*), five male (CON1, CON2, CON4, DL1, DL2) and two female (CON3, DL3), 3.16–7.44 kg (26.5–51 months old) at the beginning of behavioural training, participated in this study. All procedures were conducted under the authority of UK Home Office personal licenses and a project license held by the authors. The monkeys were housed socially in troops, separated by sex, in indoor enclosures attached to standard caging. Water was always available *ad libitum* in the home enclosure; each monkey's daily food ration was delivered in the test box and was supplemented with fruit and forage mix in the home enclosure. Three of the monkeys (CON1, CON2, DL1) underwent pretraining and then learned several two-choice visual discrimination problems in a touch-screen apparatus (Baxter & Gaffan, 2007) before beginning training on the strategy implementation task; they then acquired the scene memory task. Monkey DL2 underwent pretraining, learned two-choice visual discrimination problems (Baxter & Gaffan, 2007), then began training on the strategy implementation task. This training was discontinued after about 80 sessions in the first phase because he would not reliably complete sessions; at this point he was taught the scene task, then returned to strategy implementation training once his training on scenes was complete, at which time his performance was more reliable. The remaining three monkeys (CON3, CON4, DL3) underwent pretraining, then learned the scene memory task followed by the strategy implementation task.

At the completion of preoperative training and a preoperative performance test on strategy implementation and scene learning, three monkeys (cases DL1–DL3) received surgical ablation of the DLPFC, bilaterally in a single stage, and four (cases CON1–CON4) were retained as unoperated controls. The DLPFC lesion was limited primarily to area 46 (Petrides & Pandya, 1999) in case DL1, but was more dorsally and medially extensive in cases DL2 and DL3. The preoperative and postoperative performance tests were identical for all seven monkeys. Although all comparisons for these tasks could be made on a within-subjects basis, the presence of unoperated controls confirmed that the concurrent testing in multiple behavioural tasks produced stable measures of performance. The unoperated controls also served as a comparison for further tests carried out in between-subjects designs after the completion of postoperative testing in strategy implementation and scene learning.

### Apparatus

Behavioural testing took place in an automated apparatus. Each monkey was taken from the home enclosure into the test cubicle in a wheeled transport cage, which was fixed in front of a video-display unit with a touch-sensitive screen (380 × 280 mm, 800 × 600 pixel resolution). The monkey could reach through horizontally oriented bars (approximately 45 mm apart) at the front of the cage to reach the screen and the rewards. Stimulus presentation, recording of touches to the screen and reward delivery were all under computer control. A pellet dispenser delivered 190-mg banana-flavoured or sugar pellets (P. J. Noyes, Lancaster, NH, USA) into a food cup located below the touch-screen. Pellet delivery produced a click from the pellet dispenser as well as a 500-ms tone from the computer. A metal ‘lunchbox’ (approximately 200 × 100 × 100 mm) was located to the left of the food cup, and was filled with a mixture of wet monkey chow, seeds, apple, banana, orange, nuts and dates. Infrared cameras positioned at different locations within the test cubicle permitted observation of the monkey while it was performing the task. The entire apparatus was located in an experimental cubicle that was dark except for the illumination of the video screen.

### Behavioural testing: pretraining

The monkeys that had experience with a discrimination learning task in the touch-screen had no further pretraining before beginning training on the strategy implementation task (described in the next section). The remaining monkeys were shaped to enter the transport cage from their home enclosure, and once they were reliably taking food in the test cubicle, pretraining began. First, reward pellets were delivered on a variable-interval (2-min) schedule to accustom them to take pellets in the test box. After several days of pellet training, the touch-screen was activated and the screen was filled with an array of different-coloured alphanumeric characters on a black background (in a different size and typeface than those used in the main task). Touches to any location on the screen resulted in pellet delivery. In the third stage, single alphanumeric characters were presented in random locations on the screen, and remained until touched; a touch caused the character to disappear and a reward pellet to be delivered. Gradually, the complexity of the display was increased by introducing additional visual elements (a coloured background, coloured ellipse segments and a single large alphanumeric character). When monkeys were reliably completing 50 trials in a single test session with minimal accuracy errors (i.e. touching any location on the screen other than the small alphanumeric character) they began training on the scene memory task. The monkeys with discrimination learning experience underwent this third stage of pretraining between acquisition of the strategy task and the scene task.

### Object-in-place scene learning

The object-in-place scene learning task was adapted from Gaffan (1994). This task employed artificially constructed background scenes that occupied the whole area of the display screen. The background scenes were generated by an algorithm based on a random number generator. Each scene was unique in that it varied in several randomly selected attributes, including: (a) the background colour of the screen; (b) the location of ellipses on the screen; (c) the colour, size and orientation of ellipse segments; (d) the typographic character, clearly distinct in size from the foreground objects; and (e) the colour of the typographic character. All the colours were assigned with the constraint that the foreground objects should be visible (that is, there was a minimum separation in colour space between the colours of a foreground object and the colour of any element of its local background). Two background objects, small randomly-chosen and coloured typographic characters, were placed within each scene. In each scene, one of the two foreground objects was the correct one for the monkey to touch (rewarded) and the other was incorrect (unrewarded). The locations and identities of the foreground objects were fixed within each scene but varied between scenes. Because these scenes were randomly generated, an infinite number of unique scenes could be presented. For example stimuli, see Browning *et al.* (2005) and Gaffan (1994). After each monkey learned to touch single foreground objects against a black background, additional scene elements were introduced in shaping programs until the monkey reliably touched the foreground object when presented with a new scene. Problems were then introduced with two foreground objects (one correct and one incorrect, as described above), and the number of scenes given in each session was gradually increased, based on each monkey's performance. Training continued until performance was stable (for all seven monkeys, mean of 69.7 sessions, range 21–111).

In the final version of the task, 20 new scenes were presented in each session; the list of 20 scenes was repeated eight times. Each trial began with the presentation of a scene problem on the screen (a background scene containing two foreground objects). A touch to the correct object caused the object to flash for 2.4 s, then the screen blanked and a reward pellet (190 mg; P.J. Noyes, Lancaster, NH, USA) was delivered, followed by a 5-s intertrial interval. A touch to the incorrect object caused the screen to blank immediately, followed by a 20-s intertrial interval. Touches anywhere else in the scene caused the screen to blank and the trial was repeated, following a 20-s intertrial interval. For the first repetition of the list of scenes only, incorrect responses were followed by a correction trial in which the scene was re-presented with only the correct object present. The subsequent seven repetitions of the list of scenes did not contain correction trials, and the scenes were presented in the same order in which they were encountered in the first run through the list. Monkeys learned which object in each scene was correct by trial and error, generally very rapidly during the first run through the list, because error rates were very low during the second run through the list (13–27%; chance is 50%). When the monkey completed the final trial of a session the lunchbox opened, and the monkey received the large food reward. If the final trial was incorrect, a correction trial was given so that the monkey only ever received the large food reward following a correct response. The dependent measure was the number of errors (initial touches of the incorrect foreground object) in each presentation of the list of 20 scenes.

### Strategy implementation task

This task is identical to that described by Gaffan *et al.* (2002), except that clip art stimuli were used instead of compound alphanumeric characters. The strategy implementation task required monkeys to learn about two categories of objects. Each category was associated with a different strategy that had to be performed to obtain food reward, deemed ‘persistent’ and ‘sporadic’. Efficient performance of the task required alternation of choices between persistent and sporadic objects, with the switch occurring when reward had been earned for selection of one category. Monkeys learned the task using four pairs of objects, each pair containing one item from each of the two categories. These four pairs of objects were used throughout all preoperative and postoperative testing.

A pair of objects appeared on the touch-screen on each trial, containing one object from each category, and the monkey was allowed to choose one of the two objects. The left–right position of the objects on the screen was randomized across trials. After one of the two objects was touched, the screen blanked for a 5-s intertrial interval before the next trial was presented. Monkeys could earn rewards in one of two ways. First, four consecutive choices of the ‘persistent’ object within each pair resulted in delivery of a 190-mg pellet upon the fourth persistent choice. Second, any time after receiving a reward for choosing four persistent objects in a row, a single choice of an object from the second category (‘sporadic’) resulted in banana pellet delivery, but another sporadic reward was not given until another persistent reward had been earned. Thus, monkeys were required to alternate between choices of persistent and sporadic objects, and had to execute different behavioural strategies in order to obtain rewards from the objects in the two categories. The dependent measure was the trials/reward ratio. The choice sequence that would optimize the rate of reward delivery was for the monkey to choose the persistent object in the pair on four consecutive trials, then the sporadic object on the following trial, and then to repeat this sequence of choices, resulting in two rewards for every five trials (a trials/reward ratio of 2.5). Failing to choose the sporadic object immediately after receiving a reward for choosing four persistent objects in a row, interrupting chains of persistent responses with choices of sporadic objects, or continuing to choose the sporadic object before another reward had been earned for choosing persistent objects all contributed to less-than-optimal performance and an elevation of the trials/reward ratio. In each test session, monkeys chose objects across trials until they had earned 50 rewards. The last reward earned in each session also resulted in opening of the ‘lunchbox’ and delivery of the single large food reward.

Training procedures were identical to Gaffan *et al.* (2002) and proceeded in five phases. Briefly, monkeys were trained on this task by presenting one pair of objects at a time (containing one persistent object and one sporadic) until the trials/reward ratio was 2.94 or lower in each of two consecutive sessions in which 50 total rewards were earned, or until a total of 6000 (first problem) or 4000 (all other phases) rewards had been earned. Once this criterion was achieved with each pair individually, in the fifth and final phase (the final version of the task) the four pairs of objects were presented randomly intermixed across trials so that choice behaviour had to be guided by the category membership of each object rather than a sequence of specific object choices. Training in this phase continued to the same criterion (two consecutive sessions with a ratio of 2.94 or better or 4000 rewards earned, about 80 sessions of training). Choice behaviour was above chance in the first session with intermixed problems, mean trials/reward ratio = 4.17; chance performance would be 16.3 (Gaffan *et al.*, 2002). Monkeys that did not reach the 2.94 trials/reward criterion and advanced based on the cumulative number of rewards earned within a phase (CON2, third problem and final phase, CON4, final phase) performed comparably in their preoperative performance test to other monkeys that had achieved the criterion during training. For all seven monkeys, the mean number of sessions required to complete all five phases of training was 179 (range 77–414); to complete the final phase of training it was 43.6 (range 5–149).

### Performance tests

After completion of training on the scene learning and strategy tasks, all monkeys were given a preoperative performance test consisting of 24 sessions. The first session was scene learning, followed by five cycles of two sessions of strategy performance followed by two sessions of scene learning, then two sessions of strategy performance, then a final session of scene learning. The sequence of sessions was thus STTSSTTSSTTSSTTSSTTSSTTS, where ‘S’ represents a session of scene learning and ‘T’ represents a session of strategy implementation testing. Data from the first four sessions were not considered, to allow monkeys a period of time to adjust to the alternating tasks or to become reaccustomed to testing after surgery or rest, leaving 20 sessions of performance data (10 of scene learning, 10 of strategy implementation). In this double-alternation design we could compare performance on each task when it was preceded by performance on the same or a different task, although we did not observe any systematic variation in performance related to this variable either before or after surgery. This test was repeated in the same way beginning at least 2 weeks after surgery (for monkeys in the dorsolateral group) or an equivalent period of rest for control monkeys.

### Surgery

Neurosurgical procedures were performed in a dedicated operating theatre under aseptic conditions. Each operated monkey's neurosurgical procedure consisted of a bilateral ablation of the DLPFC. Monkeys were 34 months old (case DL1), 43 months old (case DL2) or 46 months old (case DL3) at the time of surgery; the DLPFC is functionally mature in monkeys of these ages, at least as regards its involvement in spatial working memory (Alexander & Goldman, 1978). In cases DL2 and DL3, steroids (methylprednisolone, 20 mg/kg) were given i.m. the night before surgery, and three doses were given 4–6 h apart (i.v. or i.m.) on the day of surgery, to protect against intraoperative oedema and postoperative inflammation. Case DL1 received intravenous dexamethasone (1–2 mg/kg) i.v. twice during the surgery and again (1 mg/kg i.m.) the morning after. Each monkey was sedated on the morning of surgery with both ketamine (10 mg/kg) and xylazine (0.5 mg/kg), i.m. Once sedated, the monkey was given atropine (0.05 mg/kg) to reduce secretions, antibiotic (amoxicillin, 8.75 mg/kg) for prophylaxis of infection, opioid (buprenorphine 0.01 mg/kg i.v., repeated twice at 4–6-h intervals on the day of surgery, i.v. or i.m.) and non-steroidal anti-inflammatory (either meloxicam, 0.2 mg/kg, i.v. or carprofen, 4 mg/kg, i.m.) agents for analgesia, and an H2 receptor antagonist (ranitidine, 1 mg/kg, i.v.) to protect against gastric ulceration as a side-effect of the combination of steroid and non-steroidal anti-inflammatory treatment. The head was shaved and an intravenous cannula put in place for intraoperative delivery of fluids (warmed sterile saline drip, 5 mL/h/kg). The monkey was moved into the operating theatre, intubated, placed on barbiturate (DL1, thiopentone sodium, i.v., to effect), isoflurane (DL2, 1.5–2.5%, to effect, in 100% oxygen) or sevoflurane (DL3, 2.5–4.25%, to effect, in 100% oxygen) anaesthesia. Monkeys DL2 and DL3 were mechanically ventilated; DL1 was manually ventilated as necessary during surgery to maintain normocapnia. Adjustable heating blankets allowed maintenance of normal body temperature during surgery. Heart rate, oxygen saturation of haemoglobin, mean arterial blood pressure, end tidal CO_2_, body temperature and respiration rate were monitored continuously throughout surgery.

The monkey was placed in a head-holder and the head cleaned with alternating antimicrobial scrub and alcohol, and draped to allow a midline incision. The skin and underlying galea were opened in layers. The temporal muscles were retracted as necessary to expose the skull surface over the intended lesion site. A bone flap was turned over the frontal lobes and the craniotomy was extended with rongeurs as necessary. The dura was cut and reflected over the frontal lobes. In cases DL2 and DL3, the DLPFC was removed bilaterally extending from the ventral lip of the principal sulcus medially to the dorsal lip of the cingulate sulcus, including both banks of the principal sulcus. The posterior limit of the lesion on the lateral surface of the frontal lobe followed an approximate line joining the tips of the ascending limb of the arcuate sulcus and the posterior end of the principal sulcus. The entire arcuate sulcus was to be spared. From the anterior-most point of the ascending limb of the arcuate sulcus the boundary extended vertically and then down into the interhemispheric fissure. The anterior limit of the lesion was a line extending vertically from the anterior tip of the principal sulcus, down into the interhemispheric fissure. All of the cortex was removed within these limits. In case DL1, the lesion was limited to the dorsal and ventral banks of the principal sulcus. Cortical tissue was removed by subpial aspiration using a small-gauge sucker insulated everywhere except at the tip; electrocautery was applied to remove the pia mater and control bleeding encountered during the ablation.

When the lesion was complete, the dura was sewn over the lesion site, the bone flap replaced and held with loose sutures, and the skin and galea were closed in layers. The monkey was removed from the head-holder and anaesthesia discontinued. The monkey was extubated when a swallowing reflex was observed, returned to the home cage, and monitored continuously until normal posture was regained (usually within 10 min). Treatment with analgesics and antibiotics continued following surgery in consultation with veterinary staff, for 3–5 days. Operated monkeys rejoined their social groups as soon as practicable after surgery, usually within 3 days of the operation.

### Reinforcer devaluation testing

This task followed procedures described by Málková*et al.* (1997) and Baxter *et al.* (2000) except that it took place in an automated apparatus instead of a manual one. Tests of concurrent object–reward association learning and reversal learning (in which the operated monkeys were unimpaired) were given after the completion of the strategy/scenes performance test and reinforcer devaluation testing. After completion of these tests, monkeys learned 60 discrimination problems between pairs of clip art objects. Each pair consisted of a correct (rewarded) object and an incorrect (unrewarded) object. Thirty of the rewarded objects resulted in delivery of a half-peanut, and the other 30 resulted in delivery of an M&M. The intertrial interval was 30 s regardless of whether the choice was correct or incorrect. The large food reward (as in scene learning and strategy implementation) was handed to the monkey at the end of the session, rather than being given from the metal lunchbox. Training continued until a criterion of 270 or more correct responses over five consecutive sessions (90% or greater correct) was reached. At this point a series of sessions of critical trials was presented in which the 60 rewarded objects were randomly assigned to create 30 pairs of critical trials, each offering a choice between a peanut-rewarded object and an M&M-rewarded object. Some sessions of critical trials were preceded by a devaluation procedure in which the monkey was allowed to consume one of the two food rewards to satiation before beginning the critical trial session. For the devaluation, the monkey was moved into the transport cage and remained in the housing room. A plastic box was affixed to the front of the cage containing a known amount of food reinforcer (either M&Ms or peanuts). The monkey was left undisturbed for 15 min to consume the food. If the food was completely eaten the box was refilled. The monkey was then observed closely and, once it had not taken any food for 5 min, the box was removed from the cage. Once the monkey's cheek pouches were not visibly full of food, it was moved to the testing cubicle and the critical trial session begun. The sequence of critical trial sessions was: baseline, peanut devaluation, baseline, M&M devaluation, and was repeated once. Each critical trial session was separated by at least one standard training session, and monkeys had at least 2 days of rest following a critical trial session in which devaluation occurred. The critical measure was a score composed of the difference in number of choices of objects paired with a particular food on baseline sessions and the session in which that food was devalued. These scores were added together for each devalued food, and were calculated separately for each sequence of critical trial sessions (two baseline sessions and one devaluation session with each reward), with the mean taken as the overall score. For example, a monkey that chose 12 M&M objects and 18 peanut objects in the baseline sessions (mean of the two baseline sessions), then chose five peanut objects when peanuts were devalued and seven M&M objects when M&Ms were devalued, would have a difference score of (18−5) + (12−7) = 18. If he chose 14 M&M objects and 16 peanut objects in baseline sessions of the second set of critical trial sessions, then three peanut objects and seven M&M objects when each was devalued, this would give a score of 20 for the second set of critical sessions and a difference score of 19 overall. Monkey CON3 did not participate in reinforcer devaluation testing because she would not eat the half-peanut rewards in the test cubicle.

### Histology

After completion of behavioural training each monkey was sedated with ketamine (10 mg/kg), deeply anaesthetized with intravenous barbiturate and transcardially perfused with 0.9% saline followed by 10% formalin. The brain was cryoprotected in formalin-sucrose and then sectioned coronally on a freezing microtome at 50 μm thickness. A 1-in-10 series of sections through the area of the lesion was mounted on gelatin-coated glass microscope slides and stained with Cresyl violet. Sections were inspected through a microscope and the areas of damage plotted on drawings of brain sections from a rhesus monkey brain atlas (Szwarcbart, 2005).

## Results

### Extent of DLPFC ablations

**Fig. 1:**
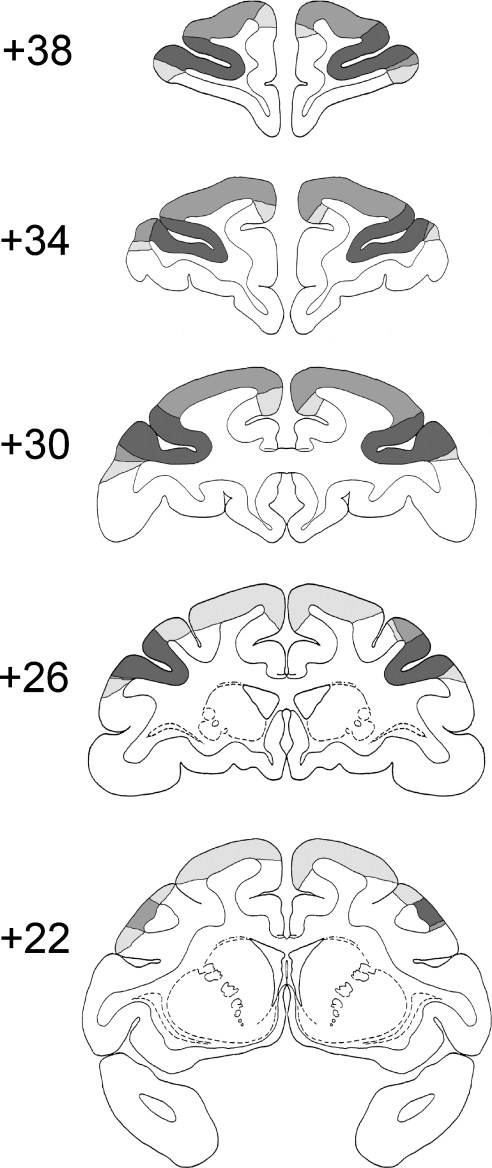
The extent of DLPFC damage is shown on coronal sections from a standard rhesus monkey brain atlas (Szwarcbart, 2005) at five stereotaxic levels through the frontal lobes at 4-mm intervals (numerals represent mm anterior to the interaural plane). Areas of lesion overlap are illustrated in shades of grey, the darkest indicating damage present in all three cases, medium indicating damage present in two of the three lesion cases, and light grey indicating damage present in only one of the three cases. Case DL1’s lesion was intended to only include the banks of the principal sulcus; this area is therefore in dark grey as it was the region common to all three cases. In the other two cases the lesion was intended to include cortex dorsal to the principal sulcus extending towards the midline but excluding the banks of the arcuate sulcus and the cortex between the arcuate sulcus and principal sulcus. The lesion extended, unintentionally, into this cortex bilaterally in case DL3 (light grey area in sections at levels +26 and +22); the posterior limit of the damage in this case is approximately level +20 mm.

The extent of the ablations is illustrated in Fig. 1, on standard sections from a rhesus monkey brain (Szwarcbart, 2005). Area 46 (as defined by Petrides & Pandya, 1999) was completely ablated in all three cases. Case DL1’s lesion was intended to include the dorsal and ventral banks of the principal sulcus only (composed primarily of area 46), and the lesion was as intended. Cases DL2 and DL3 were intended to have larger lesions that extended more dorsally and medially. The lesion in cases DL2 and DL3 used sulcal landmarks so that it was intended to remove most of area 46 and all of areas 9/46d, 9 and 8B as well as the portion of area 9/46v in the ventral bank of the sulcus principalis (areas as defined by Petrides & Pandya, 1999). This lesion also presumably included small portions of areas 10 and 8Av within the banks of the principal sulcus as well as roughly the rostral half of area 8Ad (Petrides & Pandya, 1999). The portion of area 9/46v that lies ventral to the principal sulcus was not included in the intended lesion because it is bounded inferiorly by the infraprincipalis dimple and we were not able to reliably identify this surface landmark in our monkeys. Case DL2’s lesion most closely approximated the intended lesion. The lesion in case DL3 extended posteriorly more than intended, removing cortex in areas 8Ad and 6 bilaterally that was intended to be spared. This may be related to the performance of this monkey in the scene learning task (see below). None of the lesions was intended to damage ventrolateral prefrontal cortex, ventral to the principal sulcus (Petrides & Pandya, 1999, 2002).

### Scene learning

**Fig. 2:**
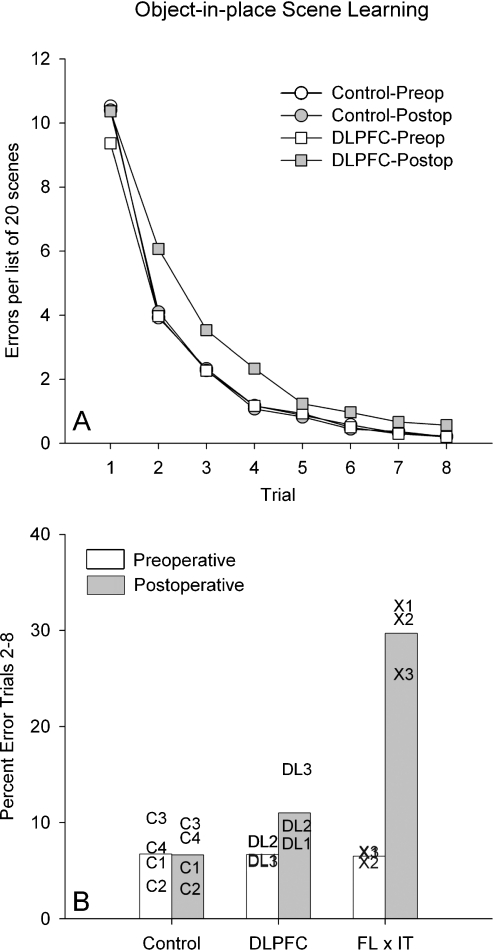
Object-in-place scene learning. (A) Learning curves across eight trials with each new scene problem; a total of 200 scenes was learned in each performance test, presented in lists of 20 scenes per session, each list being repeated eight times. Although postoperatively group DLPFC makes slightly more errors, this effect is driven mainly by a single monkey (case DL3) and is not statistically significant. (B) Individual performance scores (mean errors on trials 2–8) for each monkey. There is no overall effect of the DLPFC lesion, but case DL3, who has unintended damage to posterior frontal cortex, is mildly impaired relative to the other cases. C1–C4 represent control cases, DL1–DL3 represent individual DLPFC lesion cases. For comparison, data are shown from monkeys with frontal-inferotemporal disconnection (X1–X3; designated as A–C in Browning *et al.*, 2005) who are severely impaired postoperatively. Mean scores for pre- and postoperative tests are indicated by vertical bars.

Monkeys learned 20 new scene problems in each session. Performance on the first trial of each problem was determined by trial and error. Learning progressed rapidly after the first trial, much faster than for similar object discrimination problems presented against a neutral background (Gaffan, 1994). Bilateral ablation of DLPFC had little effect on scene learning. Case DL3, with the lesion that included unintended damage to posterior frontal cortex, was moderately impaired after the lesion, but DL1 and DL2 were hardly affected. Changes in performance between preoperative and postoperative testing were analysed by repeated-measures anova with testing phase (preop vs. postop) and each trial (repetition) of the list of scenes as within-subject factors, and lesion group (control or DLPFC lesion) as a between-subjects factor. This analysis revealed a main effect of trial, as expected, *F*_7,35_ = 342.3, *P* < 0.0005, a marginal effect of test phase, *F*_1,5_ = 4.55, *P* = 0.086, and a test phase by trial interaction, *F*_7,35_ = 2.29, *P* = 0.05. However, these effects did not interact with lesion group; test phase by lesion group, *F*_1,5_ = 5.245, *P* = 0.071, and test phase, trial and lesion group, *F*_7,35_ = 1.68, *P* = 0.146. These data are plotted in Fig. 2. Thus, there is no statistically significant effect of the DLPFC lesions on performance of this task. Figure 2A shows learning curves across the eight repetitions of lists of 20 new scenes. Figure 2B shows a summary measure (percent errors on trials 2–8 of each new list of scenes) for each monkey pre- and postoperatively. Data from monkeys with frontal-inferotemporal disconnection are shown for comparison (Browning *et al.*, 2005). A within-subjects (preop vs. postop) comparison of the summary measure of number of errors on trials 2–8 for the dorsolateral group alone also revealed no effect, *t*_2_ = 1.64, *P* = 0.24.

### Strategy implementation

**Fig. 3:**
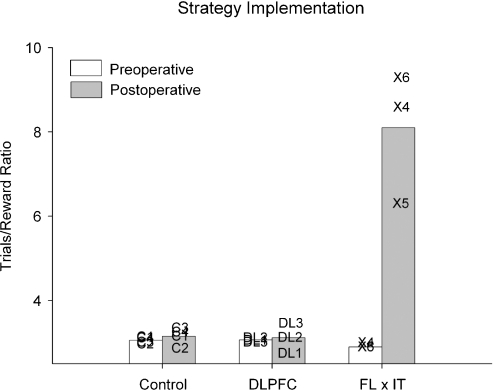
Strategy implementation performance. Trials/reward ratio is shown for pre- and postoperative performance tests (10 sessions of the strategy implementation task in each test) for controls and monkeys with DLPFC lesions. Performance on the task is unaffected by DLPFC ablation. C1–C4 represent control cases, DL1–DL3 represent individual DLPFC lesion cases. For comparison, data are shown from monkeys with frontal-inferotemporal disconnection (X4–X6; designated as S1–S3 in Gaffan *et al.*, 2002) who are severely impaired postoperatively. Mean scores for pre- and postoperative tests are indicated by vertical bars.

Dorsolateral lesions were also without effect on performance of the preoperatively learned strategy implementation task. Monkeys learned four pairs of clip art stimuli for this task, which were used in all pre- and postoperative testing on this task. One stimulus in each pair was associated with a ‘persistent’ strategy (P), the other was associated with a ‘sporadic’ strategy (S). Four consecutive persistent choices resulted in a reward after the fourth choice; any time after that a sporadic choice was rewarded immediately, but sporadic choices were not rewarded again until another persistent reward had been earned. Thus, optimal performance in this task is achieved by alternating categories of object choices upon receiving reward: make four consecutive P choices, obtaining a reward on the fourth choice, then choose S once, obtaining a reward immediately, then return to choosing P until a reward is earned again, etc. In the final version of the task the stimulus pairs were presented randomly intermixed within the test session so that performance had to be guided by the strategies associated with the objects rather than a specific sequence of choices of particular objects. The critical measure of performance was the trials/reward ratio for each session; a ratio of 2.5 represented perfect strategy implementation performance, as two rewards could be earned in five trials if the strategies were applied optimally. Changes in performance between preoperative and postoperative testing were analysed by paired *t*-tests for each group separately. Performance was stable in the control group between ‘preop’ and ‘postop’ tests, *t*_3_ = 1.03, *P* = 0.38, nor did DLPFC lesions impair performance postoperatively, *t*_2_ = 0.24, *P* = 0.42 (one-tailed). These data are illustrated in Fig. 3. Data from monkeys with frontal-inferotemporal disconnection are presented for comparison (Gaffan *et al.*, 2002).

### Reinforcer devaluation

**Fig. 4:**
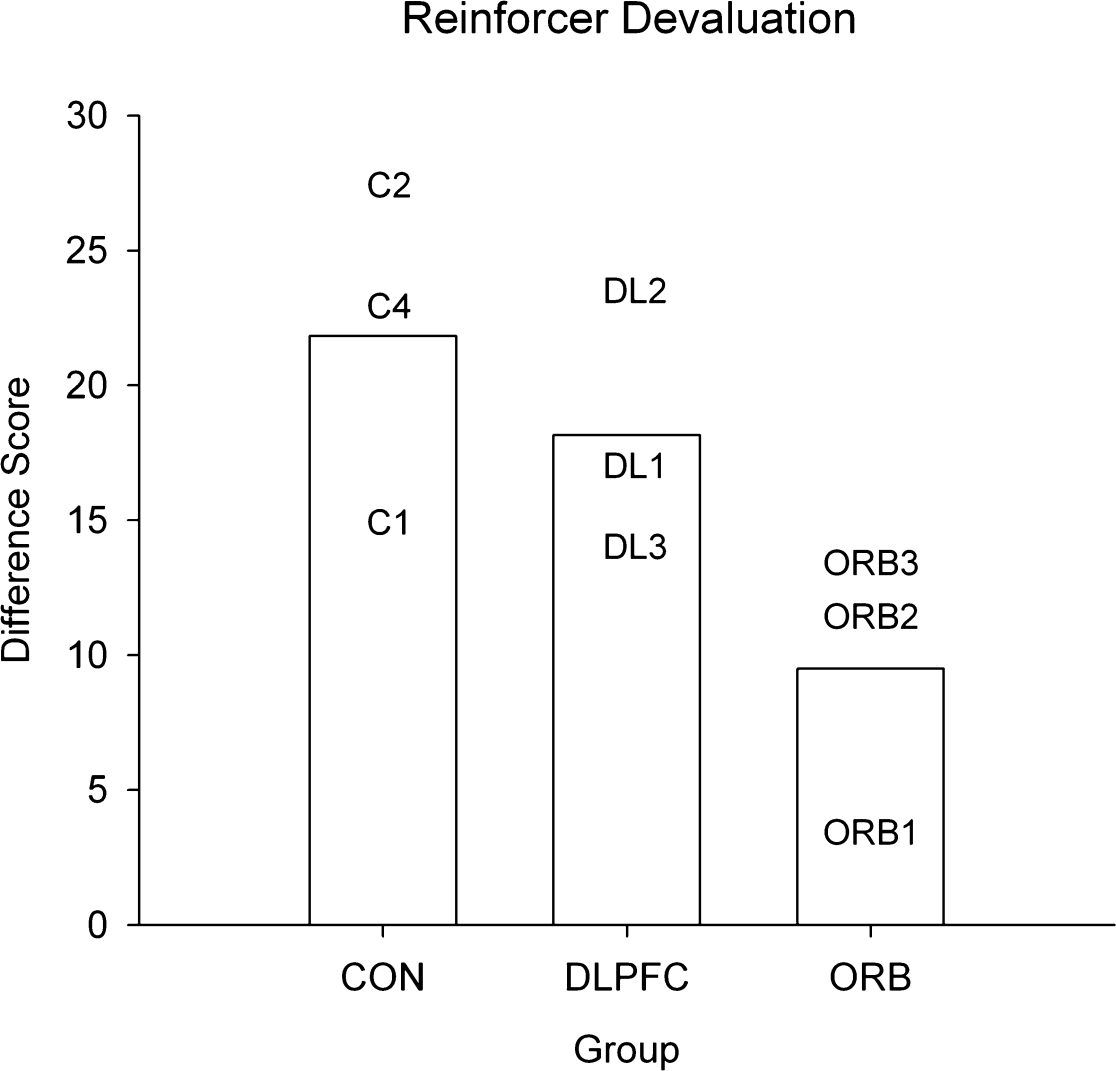
Reinforcer devaluation performance. Devaluation scores are shown, representing the difference in the number of objects chosen in baseline sessions relative to devaluation sessions in which the food associated with those objects is devalued. High scores indicate good devaluation performance, meaning monkeys adjusted their choices to avoid objects associated with the satiated food in devaluation sessions. Monkeys with DLPFC ablation achieve devaluation scores that are equivalent to those of controls. C1, C2, C4 represent control cases, DL1–DL3 represent individual DLPFC lesion cases. For comparison, data are shown from monkeys with bilateral orbital prefrontal lesions (ORB1–ORB3; M.G. Baxter, D. Gaffan, D.A. Kyriazis and A.S. Mitchell, unpublished observations) who are impaired postoperatively and were tested in the same devaluation protocol as DL1–DL3. Mean scores are indicated by vertical bars.

The two groups required a similar number of sessions to criterion to acquire the devaluation problems (control mean, 17.67; DLPFC lesion mean, 13.33), *t*_4_ = 0.74, *P* = 0.50. There was no difference in the devaluation scores for the two tests averaged together (control, 21.83; DLPFC lesion, 18.17), *t*_4_ = 0.80, *P* = 0.47, or separately, *t*_4_ < 1.42, *P* > 0.23. These data are plotted in Fig. 4. There were no significant differences in the amount of food consumed during the devaluation procedure between controls and monkeys with DLPFC lesions, for either foodstuff (*t*_4_ < 1.54, *P* > 0.20). The sensitivity of the devaluation procedure, presented in the automated apparatus, is confirmed by a significant effect of orbital prefrontal lesions on this task (M.G. Baxter, D. Gaffan, D.A. Kyriazis and A.S. Mitchell, unpublished observations; data plotted in Fig. 4).

## Discussion

Bilateral lesions of the DLPFC were without effect on three cognitive tasks that each require interaction between the frontal cortex and the temporal lobes, and engage cognitive abilities that have been ascribed to the DLPFC. Monkeys with DLPFC ablation learned new object-in-place scene problems as rapidly as unoperated controls and were unimpaired relative to their own preoperative performance. Similarly, performance of a preoperatively learned strategy implementation task was unimpaired following DLPFC ablation. Finally, DLPFC lesions were without effect on adaptive response selection in a reinforcer devaluation procedure, which required monkeys to integrate the current value of a reinforcer with information about the associations of particular visual objects with different reinforcers in order to guide choice performance.

All three cases had complete bilateral lesions of the cortex in the banks of the principal sulcus, which contains much of area 46 and whose destruction reliably impairs spatial working memory in Old World monkeys (Goldman *et al.*, 1971; Levy & Goldman-Rakic, 1999). The lesions in two of the cases extended more dorsally into additional regions of dorsal and dorsolateral prefrontal cortex. Thus, based on comparison with previously published data, the lesions in the present study would be expected to be behaviourally effective to the extent that similar lesions in other studies reliably impair spatial working memory. Admittedly, a limitation of the present study is that the behavioural effectiveness of principal sulcus damage was not verified in these three cases specifically, but it seems highly doubtful that some idiosyncratic factor common to all three cases would prevent bilateral DLPFC damage from producing impairments that would otherwise be expected.

The most reliable neuropsychological finding after DLPFC damage in macaque monkeys has been impairment in spatial working memory (Goldman & Rosvold, 1970; Goldman *et al.*, 1971; Bachevalier & Mishkin, 1986; Levy & Goldman-Rakic, 1999) and in self-ordered working memory tasks (Petrides, 1995, 2000; cf. Levy & Goldman-Rakic, 1999). Notably, none of the three tasks employed in the present study taxes spatial working memory or requires the selection of a behavioural choice from among a set of items being monitored in a working memory store. On this view, the lack of impairment in object-in-place scene learning, strategy implementation or reinforcer devaluation after DLPFC damage is not surprising. However, it is nonetheless remarkable that performance of each of these tasks, which is sensitive to damage of other subregions of prefrontal cortex – either ventrolateral, orbital, or both (Baxter *et al*., 2007, 2008; Wilson *et al.*, 2007; M.G. Baxter, D. Gaffan, D.A. Kyriazis and A.S. Mitchell, unpublished observations) – is intact after bilateral lesions of DLPFC.

New within-session scene learning was not impaired overall in the three monkeys with DLPFC lesions, although in the monkey whose lesion extended posteriorly, beyond areas 9 and 46, there was evidence of impairment. This may suggest involvement of posterior frontal regions in scene learning, but it does not implicate areas 9 and 46. This implies that, to the extent that strategic memory operations and episodic encoding and retrieval are associated with DLPFC activation (Bor *et al.*, 2003; Ranganath *et al.*, 2003; Murray & Ranganath, 2007), these capacities are not required for efficient learning of new scene problems. This lack of impairment is particularly interesting given that lesions of either ventrolateral (Wilson *et al.*, 2007; Baxter *et al.*, 2008) or orbital (Baxter *et al.*, 2007) prefrontal cortex impair scene learning, although not to the degree that frontal-inferotemporal disconnection or bilateral prefrontal lesions do (Browning *et al.*, 2005). Thus, based on the connectivity of DLPFC with these areas of prefrontal cortex, DLPFC input to orbital and ventrolateral prefrontal cortex does not seem to be required for the involvement of these cortical areas in new scene learning.

The strategy implementation task we used requires the recall of preoperatively acquired information, the sequencing and organization of behaviour in time in order to select objects from appropriate categories consistent with the reward schedules associated with them, as well as a degree of behavioural inhibition to avoid selection of inappropriate objects on each trial that will decrease the rate at which rewards can be accumulated. The reflection of progression through reward schedules and sequences of behaviour in DLPFC (Hoshi & Tanji, 2004; Ichihara-Takeda & Funahashi, 2006; Averbeck & Lee, 2007; Seo *et al.*, 2007) suggested to us that damage to DLPFC might disrupt performance of this task, but performance was unaffected following the lesions. Interestingly, performance of this task was impaired by lesions of ventrolateral prefrontal cortex (M.G. Baxter, D. Gaffan, D.A. Kyriazis and A.S. Mitchell, unpublished observations) but not orbital prefrontal cortex (Baxter *et al.*, 2007). As with scene learning, this suggests that interaction between DLPFC and ventrolateral prefrontal cortex is not required for the implementation of preoperatively learnt strategies. It is possible that lesions of DLPFC placed before acquisition of the task would have a greater effect on performance (Hampshire & Owen, 2006), even though DLPFC is not required for implementing the strategies once they have been acquired.

Performance in the reinforcer devaluation task requires monkeys to learn object discrimination problems, associate specific objects and food rewards together, and utilize information about the current value of the particular food rewards to guide their choice behaviour. It is important to note that behaviour in this task cannot be guided by learning new associations between objects and devalued foods during the critical test sessions, because each object is only encountered once in the critical test session. The convergence of information about reward preference and behavioural responses in DLPFC (Leon & Shadlen, 1999; Wallis & Miller, 2003) may suggest an important role for this area in organizing behaviour in this task, but no impairment was found after dorsolateral lesions. Again, lesions of other regions of prefrontal cortex disrupt reinforcer devaluation performance, in this case the orbital prefrontal cortex (Baxter *et al.*, 2000; Izquierdo *et al.*, 2004) but not the ventrolateral prefrontal cortex (M.G. Baxter, D. Gaffan, D.A. Kyriazis and A.S. Mitchell, unpublished observations).

To summarize, we have found that bilateral lesions of DLPFC in rhesus monkeys do not impair performance on several tests of visual memory and decision-making that have been shown to require frontal–temporal interaction, and engage some cognitive functions that have been associated with neural activity within the DLPFC. These findings support the notion of functional specialization within prefrontal cortex, for which there was until recently very little strong evidence in ablation studies (Gaffan, 2002). They may also challenge some views of prefrontal cortex in terms of hierarchical organization, by which DLPFC selects among or acts on representations in other areas, for instance ventrolateral prefrontal cortex (Wagner *et al.*, 2001) or in which prefrontal cortex is organized in terms of anterior–posterior regions (Badre & D’Esposito, 2007; Koechlin & Summerfield, 2007), all of which would be disrupted by a lesion extending through the rostral-caudal extent of DLPFC.

## Abbreviation

DLPFC: dorsolateral prefrontal cortex
